# New Insights into the Apoptotic Process in Mollusks: Characterization of Caspase Genes in *Mytilus galloprovincialis*

**DOI:** 10.1371/journal.pone.0017003

**Published:** 2011-02-11

**Authors:** Alejandro Romero, Noelia Estévez-Calvar, Sonia Dios, Antonio Figueras, Beatriz Novoa

**Affiliations:** Instituto de Investigaciones Marinas, Consejo Superior de Investigaciones Científicas, Vigo, Spain; University of Texas MD Anderson Cancer Center, United States of America

## Abstract

Apoptosis is an essential biological process in the development and maintenance of immune system homeostasis. Caspase proteins constitute the core of the apoptotic machinery and can be categorized as either initiators or effectors of apoptosis. Although the genes encoding caspase proteins have been described in vertebrates and in almost all invertebrate phyla, there are few reports describing the initiator and executioner caspases or the modulation of their expression by different stimuli in different apoptotic pathways in bivalves. In the present work, we characterized two initiator and four executioner caspases in the mussel *Mytilus galloprovincialis*. Both initiators and executioners showed structural features that make them different from other caspase proteins already described. Evaluation of the genes’ tissue expression patterns revealed extremely high expression levels within the gland and gills, where the apoptotic process is highly active due to the clearance of damaged cells. Hemocytes also showed high expression values, probably due to of the role of apoptosis in the defense against pathogens. To understand the mechanisms of caspase gene regulation, hemocytes were treated with UV-light, environmental pollutants and pathogen-associated molecular patterns (PAMPs) and apoptosis was evaluated by microscopy, flow cytometry and qPCR techniques. Our results suggest that the apoptotic process could be tightly regulated in bivalve mollusks by overexpression/suppression of caspase genes; additionally, there is evidence of caspase-specific responses to pathogens and pollutants. The apoptotic process in mollusks has a similar complexity to that of vertebrates, but presents unique features that may be related to recurrent exposure to environmental changes, pollutants and pathogens imposed by their sedentary nature.

## Introduction

Apoptosis is an essential biological process in both vertebrates and invertebrates, although its physiological function and complexity show considerable taxon-dependent variations [1]–[3]. It is critical for many aspects of animal development and also plays a key role in the maintenance of immune system homeostasis [4]. Moreover, apoptosis is considered a host defense mechanism against viral and bacterial pathogens [5], [6].

The caspase proteins constitute the core of the apoptotic machinery [7]. These proteins play two main roles: transduction of the death signal and cleavage of many cellular proteins, which results in the activation/inactivation of others and many of the biochemical and morphological changes associated with apoptosis [8]. Mutant phenotypes in mammals and flies suggest that caspases can also play important non-apoptotic roles, and the functions of some caspases are still unclear [9]. The apoptotic caspases can be categorized as initiators (apical caspases) or effectors (executioner caspases) of apoptosis, depending on where they function in the apoptotic cascade [10]. Apical caspases have long prodomains containing specific motifs such as the death effector domains (DEDs) or the caspase recruitment domains (CARDs), and mediate signal transduction and caspase activation by oligomerization. Once activated, they cleave and activate the executioner caspases [11].

Two major pathways of caspase activation have been characterized in detail in mammals. The intrinsic pathway is initiated in response to oxidative stress, genotoxic or specific signals, leading to the release of the mitochondrial cytochrome c into the cytosol [12]. Cytochrome c then activates caspase-9 family members and the caspase cascade through the apoptosome formation [13]. The extrinsic pathway is activated by extracellular ligands that are recognized by death domain-containing transmembrane receptors, resulting in the direct activation of the caspase cascade and/or induction of the mitochondrial permeability transition [14].

Genes encoding caspase proteins have been described in vertebrates and in almost all invertebrate phyla, from cnidarians to echinoderms, with major advances in the study of *Caenorhabditis* and *Drosophila* [3], [15]. The phylum Mollusca is the second most diverse group of animals after arthropods, with about 80000–150000 species living mostly in marine, brackish, freshwater and terrestrial habitats [16]. Apoptosis in mollusks is involved in the larval developmental process [17] and seems to constitute an important immune response that can be initiated by different inducers [18], [19]. Apoptosis is therefore an important cellular process in hemocytes, enabling the adequate clearance of damaged, senescent and infected cells without inflammation [20]–[22]. However, there is limited information about the genes that mediate this mechanism and their regulation under different stimuli. The caspase 8-like gene in the gastropod *Haliotis diversicolor* has been studied [23], but there are no descriptions of caspase genes in other molluscan groups such as bivalves or cephalopods.

Bivalve mollusks are highly influenced by their surrounding environment and therefore used as bioindicators in ecotoxicological studies [24], [25]. Their sedentary nature and filter-feeding behavior leads to accumulate toxic substances, such us heavy metals, polyhydroxyalkanoates (PHAs), polychlorinated biphenyls (PCBs) and pesticides, which have been reported to have immunosuppressive effects in bivalves [26]–[32]. Because bivalves are highly susceptible to climate changes, pollutants and pathogens, it could be suggested that a strong apoptotic process may be necessary to ensure body homeostasis.

In the present work, we set out to investigate caspases present in the mussel *M. galloprovincialis* (a high economical and ecological value species) and complete its apoptotic mapping. We characterized a number of novel initiator and executioner caspases and analyzed the regulation of the different pathways in response to genotoxic substances (UV light treatment and environmental pollutants) and several PAMPs.

## Results

### Characterization of caspase sequences using the RACE technique

cDNAs corresponding to the complete open reading frame (ORFs) of six mussel gene homologues to caspase proteins were obtained after RACE amplification, cloned and sequenced (GenBank accession numbers HQ424449-HQ424454). Two sequences were included into the initiator caspase group and designated as caspase-2 and caspase-8 according to their identities with the sequences available in public databases. The first sequence was 1545 bp long, encoded a protein of 468 amino acids and revealed a high identity with initiator caspase proteins such as caspase-2 from *Xenopus laevis* and caspase-9 from *Danio rerio* (E-values of 2e−16 and 2e−14, respectively). The second sequence was 1379 bp long and encoded a shorter protein of 383 residues. It was considered a caspase-8 due to its identity with caspase-8 from *Oryzias latipes* and *Tubifex tubifex* (E-values of 1e−26 and 1e−24, respectively). Four sequences were included into the executioner caspases and were named caspase-3/7-1, -3/7-2, -3/7-3 and -3/7-4 according to their identity with the caspase-3/7 group. The length of the sequences, the number of amino acids and the E-values obtained are summarized in Table 1. The identities among the executioner caspases were very low and ranged from less than 19% (between the forms -1 and -3, -2 and -3, -3 and -4), to about 50% (between -1 and -4, -2 and -4) and up to 60% (between -1 and -2).

**Table 1:** Characteristics of the mussel caspase genes included into the executioner group.

	Caspase-3/7-1	Caspase-3/7-2	Caspase-3/7-3	Caspase-3/7-4
**Total bp**	1079	1258	1340	1126
**ORF**	942	942	996	888
**AA**	313	313	331	295
**Species**	Casp3 *Bos taurus*	Casp7 *Mus musculus*	Casp3 *S. purpuratus*	Casp3 *Danio rerio*
	Casp3 *Caligus clemensi*	Casp3 *Caligus clemensi*	Casp-3/7 *Branchiostoma floridae*	Casp3 *Oryzias latipes*
**E- values**	6e−37	4e−38	4e−21	6e−32
	7e−37	5e−38	2e−19	1e−31

### Nucleotide sequence and structural analysis

**Figure 1:**
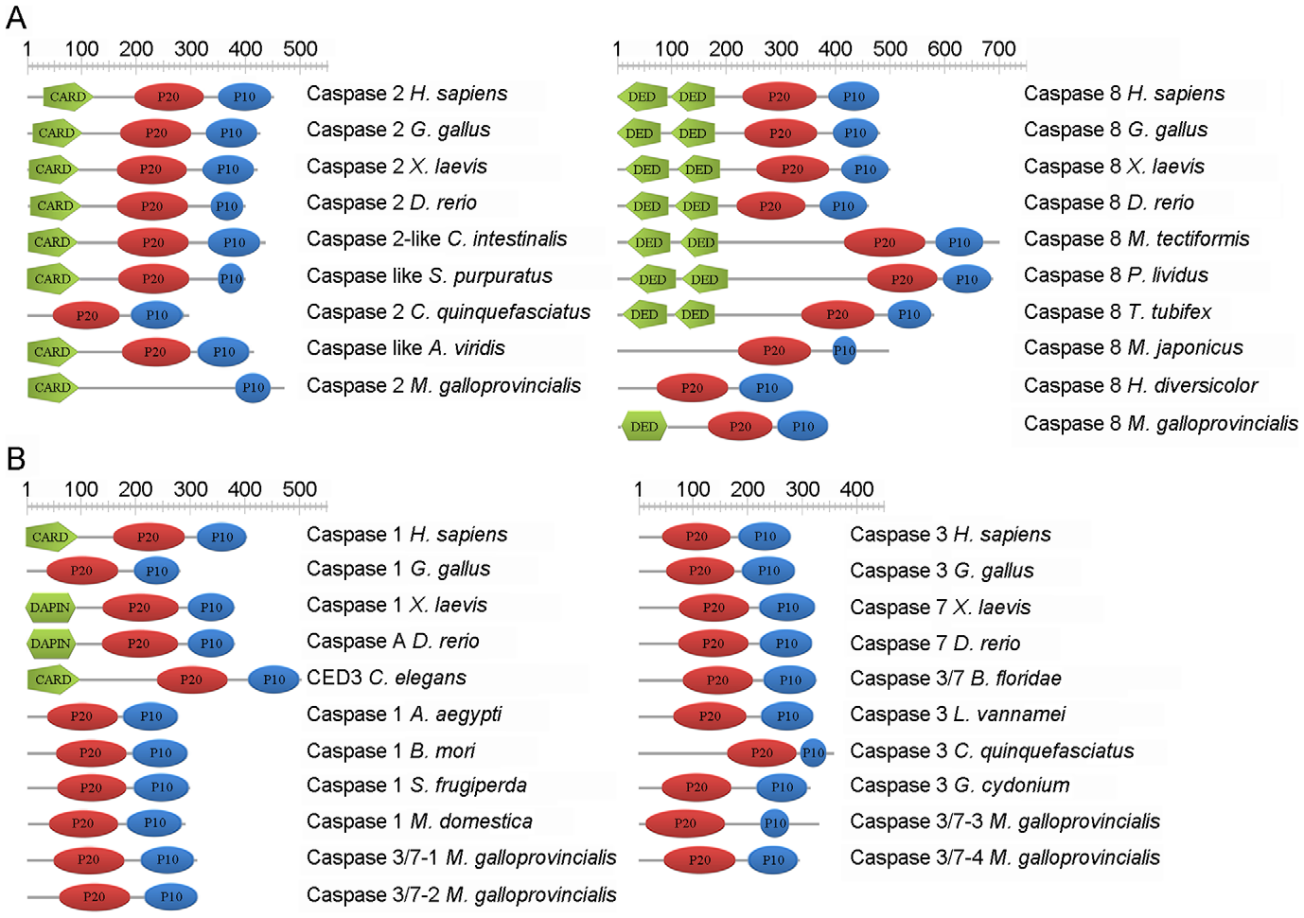
Modular architecture of initiator and executioner caspase proteins. Initiator and executioner labeled as A and B, respectively. ScanProsite was used to predict the domain features of all caspase proteins. DED domain in mussel caspase-8 was predicted by the SMART software.

**Figure 2:**
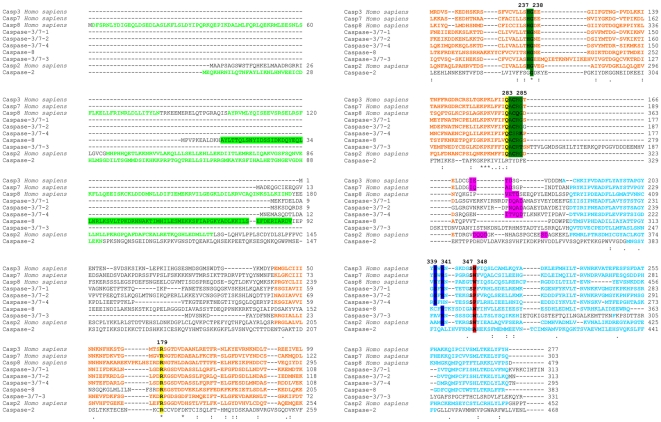
Positions of critical residues for protein structure, substrate binding and catalysis. Green letters represent the CARD and DED domains. The caspase-8 DED domain is highlighted in green. Catalytic subunits P20 and P10 are represented in orange and blue letters, respectively. Asp (D) residues at potential cleavage site between the large and small subunits were highlighted in pink. S1 site (Arg179, Gln283) is highlighted in yellow, S2 site (Ser339) and S3 site (Arg341) are highlighted in blue and S4 site (Trp348) is highlighted in red. The catalytic cysteine conserved site (QACxG) and the catalytic dyad are marked in dark green. Numbering is based on caspase-1 residue positions.

ScanProsite and SMART software was used to predict the domain features of all translated caspase sequences. Mussel caspase-2 presented one large N-terminal prodomain containing a CARD (92-residue long structural motif). SMART software predicted the presence of a single DED in caspase-8 with a low score value. Except for caspase-2, all caspase proteins presented the two subunits P20 and P10 (Figure 1A). The lack of the P20 domain in caspase-2 was obtained after several different RACE amplifications. Moreover, the structure of this molecule was confirmed by PCR amplification, cloning and sequencing of the complete molecule. The structure of the executioner caspases in mussel was similar to the structures of caspase-3, caspase-7 and caspase-1 described in insects (Figure 1B). The large and small subunits are the result of cleavage of the proenzyme at Asp (D) residues, which are homologous to human Asp316 and Asp330 (based on equivalent sites described in human caspase 2). The caspase proteins possessed four different sites called S1, S2, S3 and S4 pockets involved in the recognition of a core tetrapeptide motif (P4-P3-P2-P1) within the substrate and its catalysis. Conserved residues presented in the S1, S2, S3 and S4 sites were detected. The Arg179 and Gln283 residues from the S1 site were conserved in all caspases except for caspase-2, where the Gln283 residue was substituted. Ser339 and Arg341 characterize S2 and S3 sites, respectively. Both residues are incompletely conserved in mussel caspases. The Ser339 was replaced in caspase-2, caspase-3/7-3 and caspase-8, and the Arg341 was substituted in caspase-2 and caspase-3/7-4. The S4 binding site, which has been described as the key determinant of substrate specificity, varied markedly between the different caspases. Trp348, which helps confer the physical shape of the specificity pocket, was only conserved in caspase-3/7-3 and caspase-8. Proline replaced Trp348 (Pro, like Trp, is a hydrophobic amino acid residue) in caspases-3/7-1, -3/7-2 and -3/7-4 and Ala replaced Trp in caspase-2. The predicted active site (QACRG) was present in all caspases except for caspase-2. In a typical caspase, following substrate binding, catalysis employs a typical cysteine protease mechanism involving a catalytic dyad composed of Cys285 and His237, plus an `oxyanion hole' involving Gly238 and Cys285 (all of which were conserved in mussel except for caspase-2) (Figure 2).

### Phylogenetic analysis

**Figure 3:**
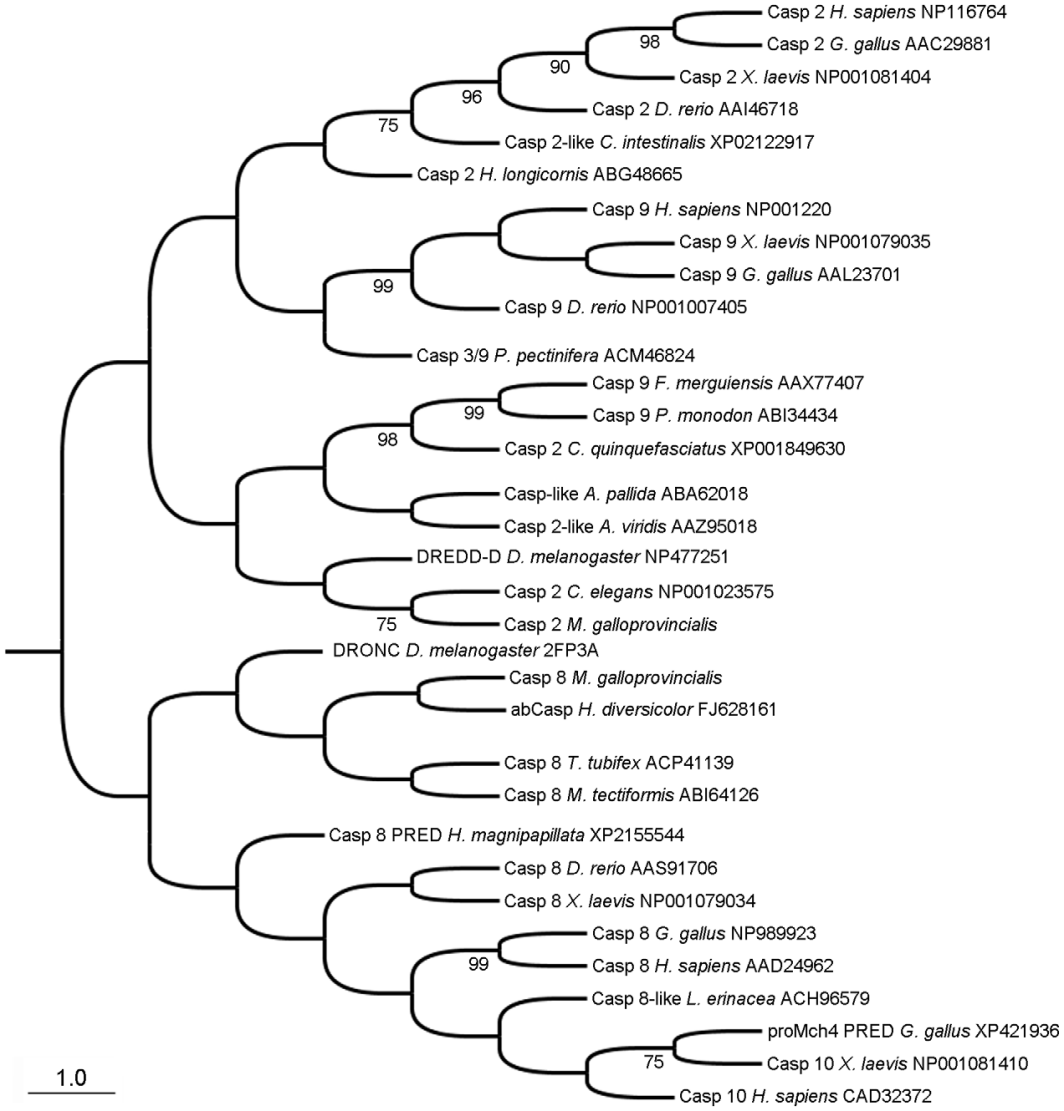
Phylogenetic analysis of initiator caspases. Neighbor-joining (NJ) phylogenetic tree for initiator caspase proteins. Numbers on branches are bootstrap percentages.

Initiator caspases were divided into two main groups (Figure 3). The first branch included caspase-2 and -9 while the second included caspase-8 and -10. Vertebrate and invertebrates caspases (-2/-9) mostly separated into other additional sub-groups. Mussel caspase-2 was placed into the caspase-2/9 main branch, close to invertebrate sequences from groups such as cnidarians, nematodes, insects and crustaceans. Mussel caspase-8 was placed into the second main branch (caspases-8/-10), far from the vertebrates but closer to the caspase described in *H. diversicolor*.

**Figure 4:**
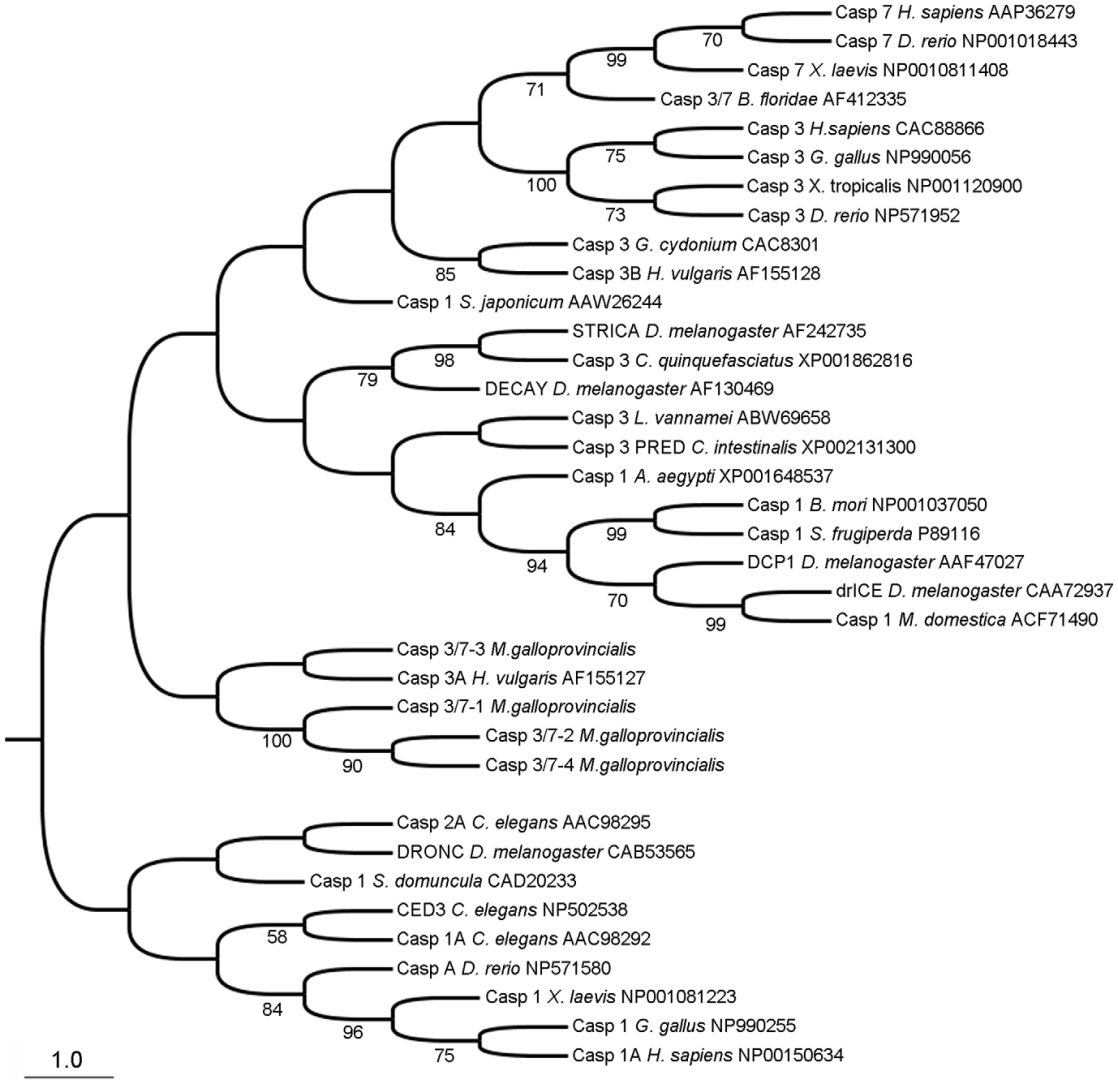
Phylogenetic analysis of executioner caspases. Neighbor-joining (NJ) phylogenetic tree for executioner caspase proteins. Numbers on branches are bootstrap percentages.

Executioner caspases from mussel were analyzed using available sequences of caspase-3, caspase-7 and caspase-1 from the GenBank database (Figure 4). Vertebrate caspase-3/7 and caspase-1 clustered into two different groups. Interestingly, caspase-1 from insects grouped together close to the caspase-3/7 branch. The four mussel caspases-3/7 were classified into one small group placed between both main branches and therefore makes up an intermediate group between the vertebrate caspase-1 and caspase-3/7 branch.

### Tissue expression analysis

**Figure 5:**
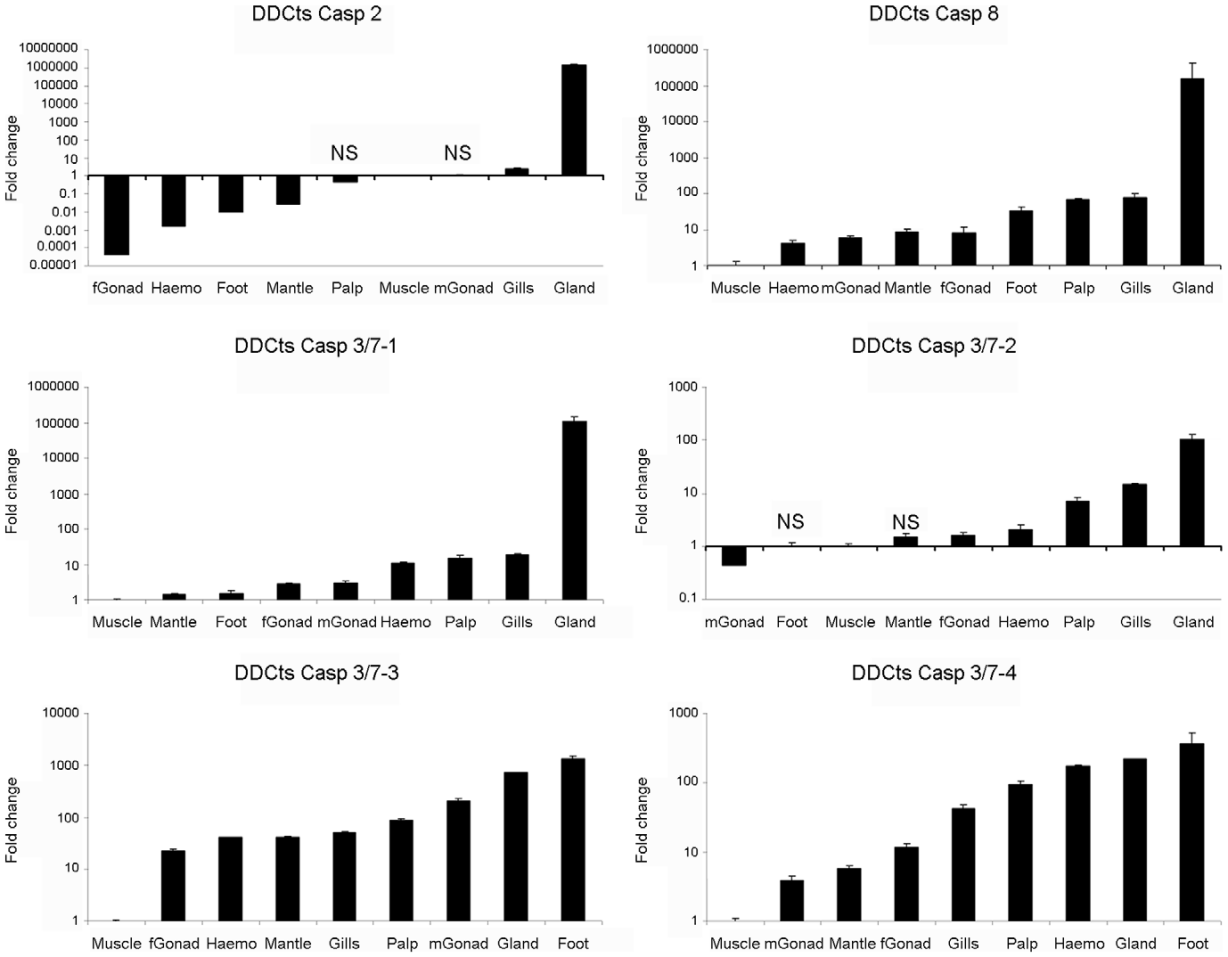
Expression of the six caspase genes in different tissues. Results represent the mean ± SD of 3 different samples. Fold change units were calculated by dividing the normalized expression values in the different tissues by the normalized expression values obtained in the muscle. Data were analyzed using the Student’s t-test. NS: no significant differences regarding to the expression levels detected in the muscle.

The analyses of caspases expression in different tissues showed that muscle tissue presented the lowest expression values in all genes except for caspase-2 and caspase-3/7-2. The lowest fold-change values for caspase-2 and -3/7-2 are found in female and male gonads (Figure 5). In general, the highest expression values for all caspase genes were detected in glands, ranging from 95 times greater than muscle for caspase-3/7-2 to 10^6^ times for caspase-2. Other tissues such as gill, foot, palps and hemocyte showed intermediate expression values and the mantle and gonads showed the lowest fold-change values. The expression values for all the caspases in the tissues sampled were significantly different from the values obtained in the muscle. Only the expression values of caspase-2 detected in the palps and male gonad and the expression values of caspase-3/7-2 detected in foot and mantle were not significantly different from the expression values obtained in the muscle.

### Apoptosis induction by UV treatment

#### Evaluation of the apoptotic process by optical microscopy

**Figure 6:**
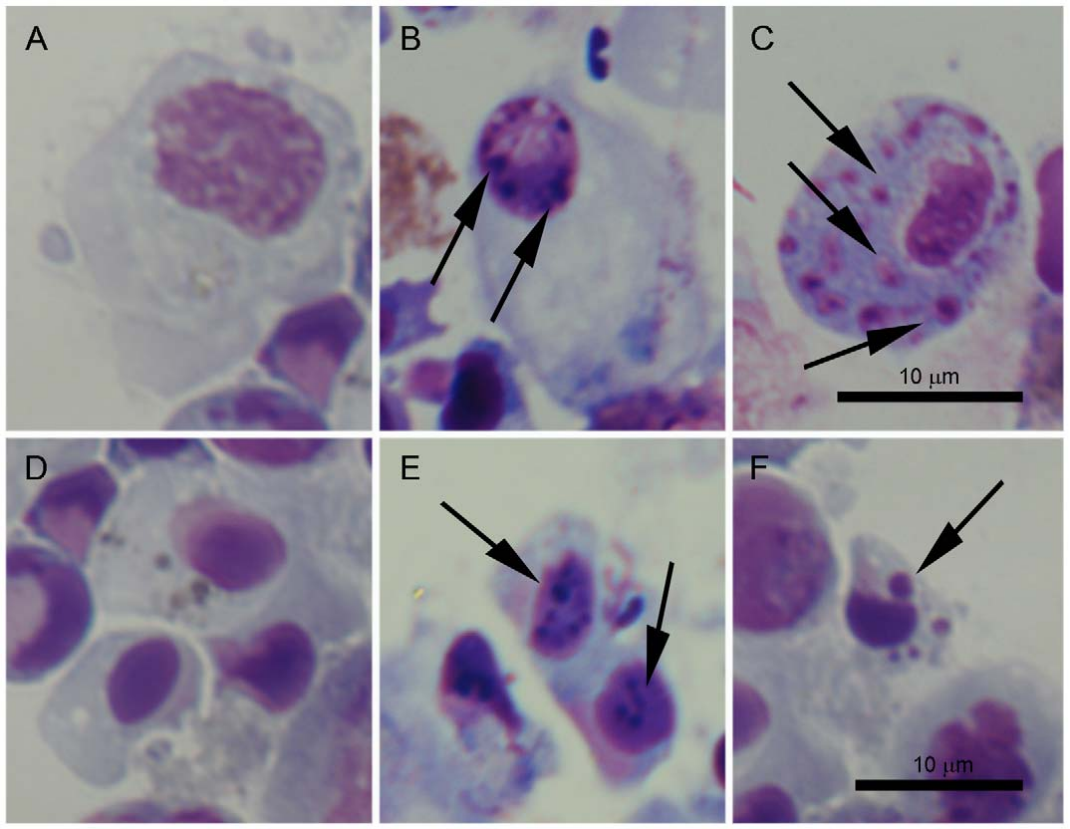
Histological changes related to the apoptotic process observed in hemocytes treated with UV light. A and D: Control granulocytes and small hyalinocytes not treated with UV-light. B: Slight chromatin condensation in granulocytes observed after 3 h pt. C and F: Appearance of intracellular bodies, positive stained for DNA, inside the cytoplasm of granulocytes and hyalinocytes, respectively after 24 h pt. E: Chromatin condensation in small hyalinocytes after 6 h pt.

Histological changes related to the apoptotic process were observed in hemocytes treated with UV-light (Figure 6). A sequential increase in morphological changes was observed during the time course. As early as 3 h post treatment (pt), a slight chromatin condensation was observed. Due to the small size of hyalinocytes, this characteristic was more clearly observed in granulocytes (Figure 6B). No morphological changes were observed at 0 and 1 h pt (data not shown). After 6 h pt, morphological changes were more evident and many dense particles were observed inside the nuclei in both types of hemocytes (Figure 6E). Apoptotic changes were highly evident after 24 h pt, when cytoplasmic vacuolization and intracellular bodies were present. These intracellular bodies were positive stained for DNA and appeared to be nuclear fragments commonly seen inside the apoptotic cells (Figures 6C and 6F). None of the related histological changes were detected in the control groups (Figures 6A and 6D).

#### Evaluation of the apoptotic process by flow cytometry

**Figure 7:**
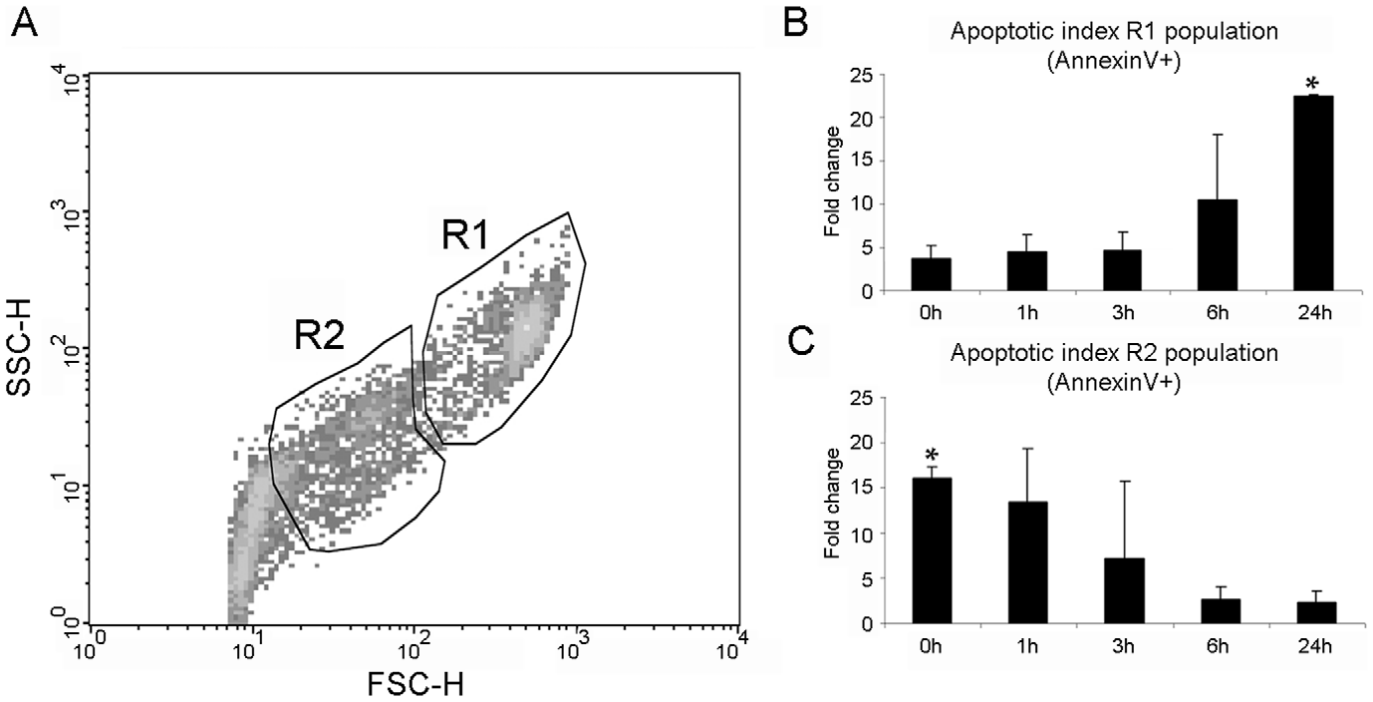
Evaluation of apoptosis induced in hemocytes treated with UV light by flow cytometry. A: Hemocytes were divided into two populations according to their FSC and SSC characteristics. B and C: Changes of apoptotic levels during the time course in R1 and R2 populations, respectively. Results represent the mean ± SD of data from 9 hemocyte populations. Data were analyzed using the Student’s t-test. * Indicates significant differences (p<0.05).

Apoptosis in different populations of hemocytes was analyzed by double-staining (AnnexinV-FITC/7AAD) flow cytometry. Based on differential density plots, total hemocytes were grouped into two main populations. The R1 population with the highest FSC and SSC values grouped mainly granular cells, meanwhile the R2 population with lower FSC and SSC values grouped small hyalinocytes (Figure 7A). A small number of R1 cells were positive for AnnexinV staining after 0, 1 and 3 h post UV-treatment, although the number of positive cells (AnnexinV+) was 5 times higher in UV-treated cells than in the controls. An incremental increase in the number of R1 positive cells during the time course was observed. A significant increase in the number of positive cells was observed after 24 h pt when compared to the results obtained at the beginning of the experiment. At this time point, the number of positive cells was 23 times higher in UV-treated cells than in the control group (Figure 7B). Trends were reversed for the R2 region. The highest positive values were registered at 0 h pt, when the number of positive cells was 16 times higher in UV-treated cells than in the controls. The fold-change value of R2 cells at 0 h pt was significantly different from the values obtained at 6 and 24 h pt (Figure 7C). In all populations treated with UV-light, the percentage of necrotic cells (7AAD+ cells) was lower than 15% and did not increase during the time course (data not shown).

#### Modulation of caspase gene expression by UV treatment

**Figure 8:**
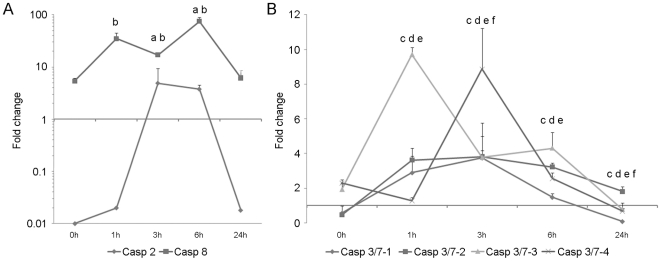
Kinetics of caspase gene expression in hemocytes treated with UV light. Results represent the mean ± SD of 3 pools (3 hemocyte populations per pool). Data were analyzed using the Student’s t-test. A: Evolution of the expression levels of initiator caspase-2 and caspase-8. a and b indicate significant differences in caspase-2 and -8, respectively. B: Evolution of the expression levels of executioner caspase-3/7-1, -3/7-2, -3/7-3 and -3/7-4. c, d, e and f indicate significant differences (p<0.05) in caspase-3/7-1, -2, -3 and -4, respectively.

The treatment of hemocytes with UV light induced important changes in caspase gene expression profiles during the time course. Caspase-2 was highly down-regulated at 0 h pt (0.01 times lower than controls) and significant increases were recorded after 3 h and 6 h pt. Expression levels decreased at 24 h to initial conditions (Figure 8A). Caspase-8 showed the highest expression values, reaching a 75-fold change maximum value over the control after 6 h pt. This gene maintained high expression levels throughout the experiment; even the lower expression values, recorded at 0 and 24 h pt were 6 times higher than the control (Figure 8A).

The expression kinetics of the executioner caspase-3/7-1 was quite similar to that of caspase-3/7-2. At 0 h pt, both caspases were down-regulated (0.5 times lower than the controls). A significant increase was then recorded after 1 h, 3 h and 6 h pt, reaching maximal levels after 3 h (3.8 times greater than the controls for both caspases). Caspase-3/7-1 was significantly inhibited at 24 h (0.08 times lower than the control); meanwhile, caspase-3/7-2 maintained the up-regulation throughout the time course and until the final measurement at 24 h pt (Figure 8B). Caspase-3/7-3 was always up regulated until 24 h. Although values significantly higher than controls were recorded at 1, 3 and 6 h pt, the maximum expression value was obtained at 1 h pt. Caspase-3/7-4 was only significantly up-regulated after 3 h pt, after which the expression value decreased to reach a significant down-regulation at the end of the experiment (Figure 8B).

### Modulation of caspase gene expression under different stimuli

**Figure 9:**
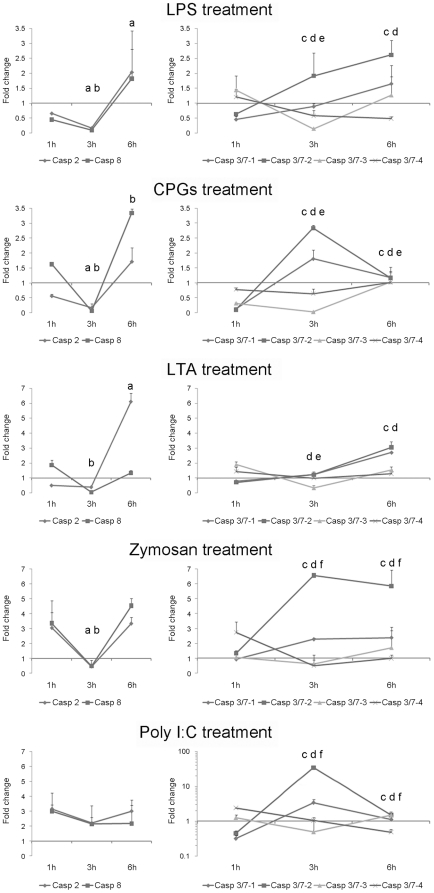
Induction of caspase genes in hemocytes treated with different PAMPs at 1, 3 and 6 h pt. Results represent the mean ± SD of 4 experimental hemocyte pools. Data were analyzed using the Student’s t-test. a, b, c, d, e and f indicate significant differences (p<0.05) of caspase-2, -8, 3/7-1, -3/7-2, -3/7-3 and -3/7-4, respectively.

In order to analyze the implication of caspases in the pathogen-induced immune response, hemocytes were treated with various PAMPs (Figure 9). The PAMPs that mimicked bacterial infections (LPS, CPGs and LTA) were able to modulate caspase gene expression. LPS and CPGs induced a less than 3.5-fold change in gene expression. Initiator caspase-2 and -8 were significantly down-regulated after 3 h pt for all treatments. By the end of the experiment (6 h pt) LPS and LTA treatments had induced a significant up-regulation of the caspase-2 gene, whereas the CPGs treatment significantly up-regulated caspase-8 expression. Executioner caspase-3/7-1 and -3/7-2 followed similar kinetics for all treatments. LPS and LTA induced an increase of both caspases during the time course, caspase-3/7-2 expression reaching levels significantly different than controls as early as 3 h pt; caspase-3/7-1 was not significantly up-regulated in LTA treated samples at the 3 h pt time point. During CPGs treatment, the expression levels of caspase-3/7-1 and -3/7-2 peaked after 3 h pt followed by a significant decrease of the expression after 6 h pt. Caspase-3/7-3 and -3/7-4 were less affected by the treatments during the time course. Caspase-3/7-3 was consistently significantly down-regulated after 3 h pt in all treatments, recovering initial expression values at 6 h pt in LPS and LTA treatments or reaching maximum values in CPGs treatment. No significant changes were recorded for caspase-3/7-4 expression.

Zymosan was also used to stimulate hemocytes because it is a component of the yeast cell wall. The expression responses obtained in hemocytes treated with zymosan were partially similar to those described for bacterial treatments. There was significant down-regulation of caspase-2 and caspase-8 expression at 3 h pt. After 6 h pt, the expression values recovered to reach levels observed at 1 h pt (3 times greater than the control group). Executioner caspase-3/7-1 and -3/7-2 were over-expressed at 3 h and 6 h; meanwhile, caspase-3/7-4 was significantly inhibited at 3 h and 6 h. No significant changes were recorded in caspase-3/7-3 expression.

Gene expression kinetics after viral infections, mimicked by the poly I:C treatment were different than in the previously described treatments. Although the changes on the expression levels of both initiator caspases during the time course were not significant, they displayed fold-increases higher than 2. Expression of executioner caspase-3/7-1 and -3/7-2 was induced to a significant level (3 and 6 h pt), reaching levels up to 34 times over their expression registered in the controls (a 34-fold increase was observed in caspase-3/7-2 expression only). However, 3/7-1 and -2 forms presented fold-change values lower than the control 1 h pt. No significant changes were recorded in caspase-3/7-3 expression while caspase-3/7-4 was significantly down-regulated at 3 h and 6 h pt.

**Figure 10:**
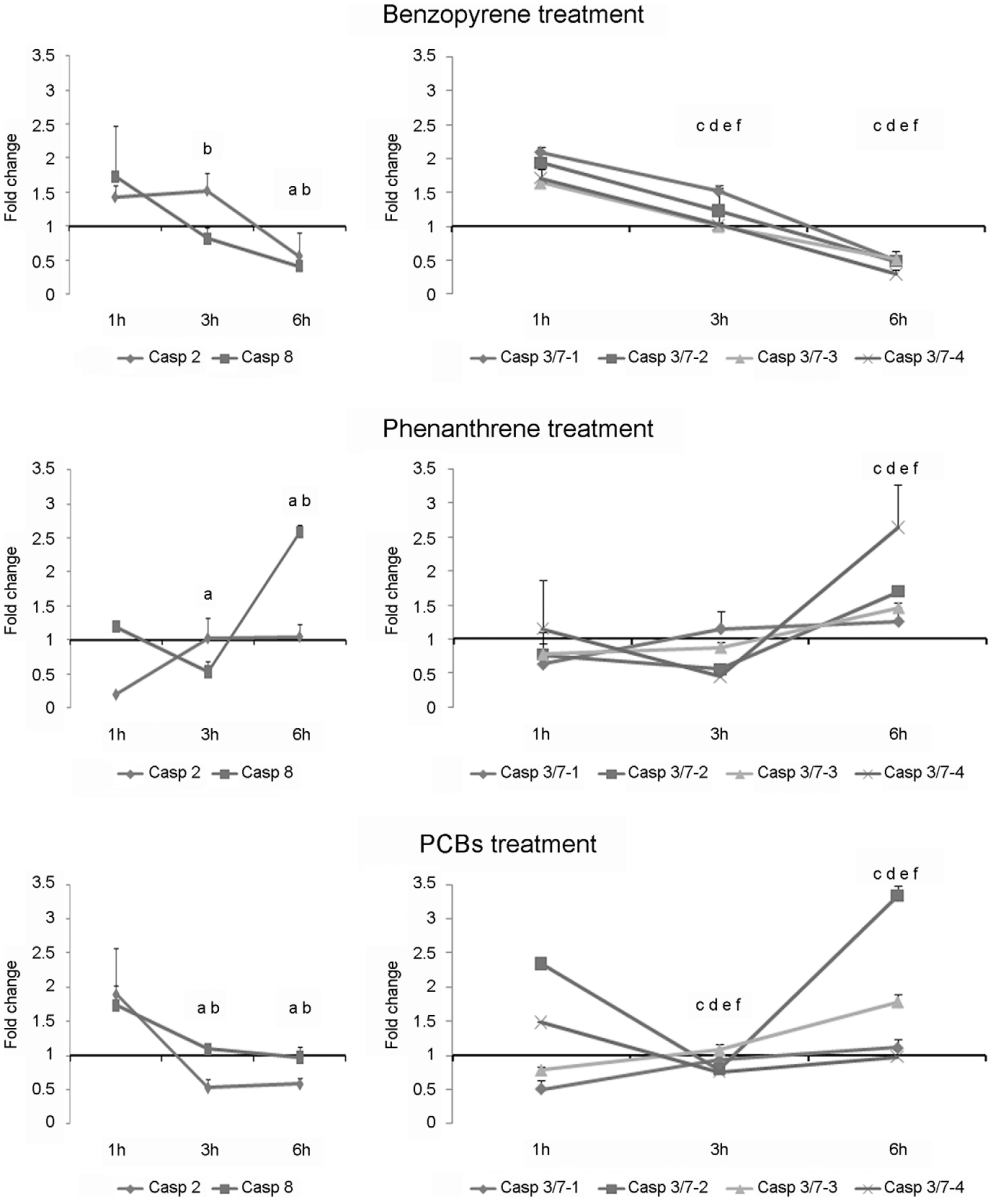
Modulation of caspase gene expression in hemocytes treated with bezopyrene, phenanthrene and PCBs. Results represent the mean ± SD of 4 experimental hemocyte pools. Data were analyzed using the Student’s t-test. a, b, c, d, e and f indicate significant differences (p<0.05) of caspase-2, -8, 3/7-1, -3/7-2, -3/7-3 and -3/7-4, respectively.

In order to evaluate the effect of different pollutants on the mussel immune system, hemocytes were exposed to high concentrations of PHAs and PCBs. These compounds are some of the most prevalent components of crude oil. Therefore, PHAs and PCBs are some of the chemicals responsible for marine environmental contamination due to oil spills. Both compounds induced changes in the gene expression profiles ranging from a 3-fold-increase to 0.3 times below the controls (Figure 10). Benzopyrene induced a significant down-regulation of all caspase genes by the end of the time course. At 1 h pt, all genes were expressed at least 1.5 times but expression decreased gradually until 6 h pt, when all the genes were expressed at levels less than controls. Opposite results were obtained for hemocytes treated with phenanthrene; the expression of all caspase genes significantly increased during the time course. Caspase-2, -3/7-1, -3/7-2 and -3/7-3 were down-regulated at 1 h pt but expression levels increased to reach levels greater than the control at 6 h pt. The fold-change expression at this sampling point was less than 2 for all genes. The expression of caspase-3/7-4 and caspase-8 increased during the course of the experiment and displayed fold-change values higher than 2.5 by the last time point. Modulation of gene expression was also observed in hemocytes treated with PCBs. Initiator caspase-2 and -8 were significantly down-regulated after 3 h and 6 h pt. Executioner caspase-3/7-1 and -3/7-3 were up-regulated significantly at 3 h and 6 h pt; caspase-3/7-4 was significantly down-regulated. Caspase-3/7-2 showed significant down-regulation after 3 h pt and significant up-regulation after 6 h pt.

## Discussion

Sokolova has highlighted the importance of the apoptotic process in the molluscan immune defense system [19]; additionally, a description of the caspase-8 gene in the gastropod *H. diversicolor* has been recently reported [23]. Despite the economical and ecological importance of bivalves, there are few reports on the description of caspase genes or the effects of different stimuli on the modulation and/or regulation of different apoptotic pathways. In the present work, we characterized 6 different caspase genes in the bivalve mollusk *M. galloprovincialis* and also analyzed their regulation under experimental exposures to genotoxic substances and several PAMPs.

Based on the BLAST results, the prediction of structural domains and the phylogenetic analyses, the translated mussel caspase sequences were classified as either initiators or executioners. The initiator caspase group was composed of 2 sequences with high identity to caspase-2 and caspase-8, whereas the executioner group was composed of 4 members with high identity to caspase-3/7. Although we characterized 6 caspase genes in the present work, the Mediterranean mussel most probably possesses additional caspase genes. The set of initiator caspases (caspase-2 and caspase-8) has high sequence similarity with the sequences found in other animal models and could be homologues to the *Drosophila* Dredd and Dronc genes, respectively [3], [33]; interestingly, there was greater variability found within the executioner caspase group. Due to the low identity values obtained among caspase-3/7-1, -3/7-2, -3/7-3 and -3/7-4, we suggest that these sequences could correspond to different proteins rather than being different isoforms of the same protein. The presence of different isoforms of executioner caspases has been described in different phyla, from humans to sponges [34]–[37], but only 3 of the 31 caspase genes described in equinoderms have been described as executioner proteins [33]. The appearance of multiple executioner caspases in bivalves could reflect a mollusk-specific expansion of these proteins, highlighting the importance of the executor phase in the apoptotic process of mollusks. Moreover, this expansion of the executioner caspases could be the reason why appeared as divergent whereas the initiator caspases appeared to be substantially conserved.

The four executioner caspases in mussel contain short prodomains and both the conserved cysteine active site pentapeptide and the typical p20-P10 domain structure well described in mammals [36]. In addition, the critical amino acid residues involved in catalysis [38] were well conserved in the four executioner caspases. However, the residues forming the binding pocket for P1 aspartic acid in the substrate were not conserved [38], the executioner caspase-3/7-3 and -4 presented two different substitutions. Since these residues play a role in determining caspase substrate specificity, the specificities of these caspases may differ substantially from those of other family members.

The most important structural characteristic of initiator caspases was the presence of the CARD and DED in caspase-2 and caspase-8, respectively. Although it has been suggested that death receptor-mediated apoptosis may not be functional in non-chordate invertebrates [19], the presence of both initiator domains in mussel, as along with previously reported descriptions of several proteins containing death domains from other invertebrates [14], [39], suggest that there is the possibility of a death receptor-mediated pathway in invertebrates. The mussel caspase-2 lacks the conserved cysteine active site pentapeptide, which indicates that it may represent a non-proteolytic caspase-like regulatory protein analogous to the mammalian protein CLARP (caspase-like apoptosis-regulatory protein) [40]. Mussel caspase-2 also lacks the typical p20 domain shared for most other caspase-2 proteins. It could be that this form of caspase-2 was a splice variant of a full length functional caspase in mussel. This seems to indicate a potential regulatory function for this truncated form of caspase-2, similar to other truncated forms of caspase-2 described in humans [41]. Mussel caspase-8 also has structural features that make it different from other caspase-8 proteins. SMART software predicted the presence of only one DED domain in mussel caspase-8 instead of two DED domains described in higher vertebrates [36], marine tunicates [42], invertebrates such us equinoderms [33] and annelids (unpublished sequence available in GenBank with accession number FJ890021.1). This DED domain was also absent in *H. diversicolor* [23] and in the marine shrimp *Marsupenaeus japonicas* [43], [44].

After analyzing the tissue expression patterns of the mussel caspase genes, we could conclude that this pattern seems to be in agreement with the physiology and function of each organ. The digestive gland showed extremely high expression values of almost all caspase genes, reaching values up to 10^6^ times over the expression value registered in muscle. The apoptotic process in the digestive gland could be quite important since it is the first entrance for potential pathogens and it correlates with a high prevalence of parasites; the presence of the copepod *Mytilicola intestinalis* was found in almost 83% of the mussel population [45], [46]. This parasite injures the intestinal epithelium of infected mussels [47]; therefore, the apoptotic process would be important for the clearance of damaged cells in order to maintain the structural integrity of the digestive epithelium. High caspase expression values were also seen in the gills, where apoptosis could play a role similar to the role in the gut. Gills are exposed to environmental conditions and pollutants, and are also affected by parasites [45], [46], [48], which can cause pathological reactions in this tissue, such as disorganization of the gill filaments, leading to a reduction of the feeding capacity of the mussel [49]. Caspase gene expression in hemocytes was also high, as previously described [18], [19], which is not surprising given that the hemocytes are the immune cells in bivalves and apoptosis has been described as a host defense mechanism against parasites, viral and bacterial pathogens [5], [6], [18]. The muscle, mantle, gonads and foot showed medium or low expression values.

In order to understand the mechanisms of regulation of the caspase genes, mussel hemocytes were exposed to different stimuli and apoptosis was evaluated by microscopy, flow cytometry and qPCR techniques.

The intrinsic pathway of apoptosis was induced by a 45-min UV light treatment; it is known that UV light activates the stress kinase pathways (JNK, MAPK), induces mitochondrial and DNA damage, and therefore caspase-mediated apoptosis, in a variety of cell cultures [50]. The microscopic observations revealed that apoptotic hemocytes undergo morphological and physiological changes such as cell shrinkage, chromatin condensation and formation of apoptotic bodies composed of membrane-bound cytoplasm, nuclear fragments and organelles, resembling those extensively described by Kerr et al. [1]. Based on the four hemocyte sub-populations, described by García-García [51] using flow cytometry, we analyzed the effects of UV light in two main regions: R1 (granulocytes) and R2 (hyalinocytes). Although the two populations were susceptible to the UV light treatment, the damages induced in the hyalinocyte population were detected earlier (0 h pt) than the damages induced in the granulocyte population (24 h pt). It seems that the cytoplasmic granules of granulocytes could have some protective effect against the UV-radiation, similar to a phenomenon described in human skin melanocytes [52]. In the initiators, the qPCR results displayed high expression values in all sample points for caspase-8. Although this caspase is mainly involved in the initiation of apoptosis by the extrinsic pathway [11], [53], this was an expected result since its participation in the intrinsic apoptotic pathway through the cleavage of BH3 interacting domain death agonist (BID) protein has been described [54]. Low levels of caspase-2 were detected at 0, 1 and 24 h pt, with greater expression levels detected at 3 and 6 h pt. Caspase-2 is known to function upstream of mitochondria permeabilization after UV irradiation. It has an implied role in the translocation of Bax to the mitochondria and subsequent release of cytochrome c and Smac/DIABLO from these organelles [41]. Full-length caspase-2 has been described to have a different function than the truncated forms of caspase-2, inducing (2L) or suppressing (2S) cell death [55]. Our results showed that mussel caspase-2 is regulated by UV light but when analyzing the observed expression patterns, its role as inductor or suppressor could not be clarified. Executioner caspases were always upregulated. Caspase-3/7-3 and caspase-3/7-4 also showed a maximum peak expression after 1 h and 3 h pt, respectively. These kinetics could reflect a sequential activation of these two forms.

Bacterial infection can trigger apoptosis by modulating caspase expression. This response has been described in almost all vertebrates, from mammals [56] to fish [57], as well as in invertebrates such us *H. diversicolor* [23] and *Litopenaeus vannamei* [58]. The stimulation of hemocytes with LPS, LTA and CPGs induced up-regulation of both initiator (-2 and -8) and executioner (-3/7-1 and -3/7-2) caspases. This situation has been previously reported in mollusks, with up-regulation of the caspase-8 gene in abalones after an experimental infection with a bacterial suspension [23], [44] and in crustaceans (*L. vannamei*), with an increase of caspase-3 expression after infection with *Vibrio alginolyticus* [58]. It is interesting to point out that there was a down-regulation in caspase-3/7-3 at 3 h pt and no important changes in the expression levels of caspase-3/7-4. This supports the idea that different executioner caspases may respond to different stimuli, which could be related to the substrate affinity based on their particular structure reported above. The modulation of caspase gene expression by viral infections has been reported in humans [59] but also in invertebrates such as lepidopteran, coleopteran species [60] and in the marine shrimp *M. japonica.* In the shrimp, the infection with white spot syndrome virus induced a significant up-regulation of caspase-3 and -8 [43]. The treatment of mussel hemocytes with polyI:C induced a decrease in the initiator caspase (-2 and-8) expression levels and also in the executioner caspase-3/7-4. The other executioner caspases (caspase-3/7-1 and caspase-3/7-2) were up-regulated, with the greatest effect after 3 h post-treatment. These results may suggest a specialization of caspase-3/7-1 and -3/7-2 in the mussel immune response against viruses. The results obtained in mussel hemocytes treated with zymosan suggested that apoptosis could be involved in protective immunity to yeast infections, similar to what is known from pathogenic fungus episodes in humans [61]. We found differences in the expression patterns of initiator and executioner caspases against several PAMPs, both between and within group of caspases; however, taking together all the results we can conclude that there seems to exist caspase-specific responses to bacterial and viral pathogens.

It has been well described that pollutants have varied effects on the mollusk immune system, inducing immunosuppression [62] and activating different cellular mechanisms such as apoptosis [29], [31]. The results obtained in the analysis of gene expression on hemocytes exposed to pollutants showed a pleiotropic effect of the treatments. The treatment with benzopyrene induced a down-regulation of all caspase genes, reflecting an immunosuppressor effect; the treatments with phenanthrene and PCBs seemed to induce the apoptotic process through the up-regulation of all caspase genes. Our results are in agreement with previous works, although the magnitude of the response was dependent on the dose and time of exposure to the pollutants [29], [31], [62].

Our results suggest that the apoptotic process in bivalve mollusks could be tightly regulated by overexpression/suppression of the caspase genes. Nevertheless, with the data reported in this work, it can not be ignored that it is difficult to distinguish between the mortality percentage due to programmed cell death and the mortality due to non-apoptotic process. Apoptosis has a similar complexity to vertebrates, but also has unique features that may be related to specific requirements imposed by mollusks’ sedentary nature and their immunological response to recurrent exposures to environmental changes, pollutants and pathogens.

## Materials and Methods

### Animals

Mediterranean mussels (*M. galloprovincialis*) obtained from a commercial shellfish farm from the Ría de Vigo (NW of Spain) were maintained in open circuit filtered seawater tanks at 15°C with aeration for 1 week prior to the experiments. Animals were fed daily with *Isochrysis galbana*, *Tetraselmis suecica* and *Skeletonema costatum*. All the animal experiments were reviewed and approved by the CSIC National Committee on Bioethics.

### Characterization of caspase sequences by RACE technique

Six different partial sequences with high homology to caspase proteins were selected from two cDNA libraries previously constructed in this species (MytiBase cluster IDs: MGC07567, MGC06860, MGC08080, MGC08080.2, MGC05753, MGC08613) [63]. Amplification of the 5’ and 3’ ends of the selected sequences was done using the BD SMART RACE cDNA amplification kit (BD Bioscience Clontech). Total RNA was isolated from hemocytes using the Trizol reagent, following the manufacturer’s instructions, and resuspended in DEPC-treated water and stored at -80°C. The quality of total RNA was measured by agarose gel electrophoresis. First, strand 3′and 5′RACE-cDNAs were synthesized from 1 µg of total RNA using BD PowerScript Reverse Transcriptase at 42°C for 90 min with 3′-CDS primer A and 5′-CDS primer and BD SMART II A oligo, respectively, following the manufacturer’s instructions. 3′and 5′-end sequences of the different caspase genes were obtained by PCR with Universal primer A mix and specific primers designed with the Primer3 (v. 0.4.0) software (Table 2). PCR reactions were performed as follows: 25 cycles at 94°C for 30 s, annealing at 68°C for 30 s, and elongation at 72°C for 3 min, followed by a 7-min extension at 72°C. Products were analyzed in 1% agarose gels stained with ethidium bromide using a GelDoc XR machine (Bio Rad) and cloned into the pCR2.1-TOPO vector (Invitrogen) using DH5α™ Competent Cells (Invitrogen). Blue/white screening of colonies was done on plates containing X-gal. White colonies were screened by PCR using M13 forward and reverse primers and positive colonies were sequenced using the same primers.

**Table 2:** Sequences of primers used for RACE amplification.

Name	Sequence
Mussel_Casp-2_RACE3-2.1	GTTTACCATATGACAGGTAGTAAGAAAGG
Mussel_Casp-3/7-1_RACE5-4	CAGGACAGTTCGTTGCATTGAAGTATTCC
Mussel_Casp-3/7-2_RACE5-1	CCATAGCATCTGCCTGGTCTGGATCTC
Mussel_Casp-3/7-2_RACE3-1	TTCAGGCCTGTAGAGGAGGAGGATTAGG
Mussel_Casp-3/7-3_RACE5-1	TCCCCAGGCCTGTCTTTGTAACCAG
Mussel_Casp-3/7-3_RACE3-1	CCAGGTGGTGTTGATGATGATGAGGA
Mussel_Casp-3/7-3_RACE3-2	CACGATTGTACGGGGCATTTTCTCC
Mussel_Casp-3/7-4_RACE3-3	TACGGGGCATTTTCTCCTGGCTGTT
Mussel_Casp-3/7-4_RACE3-4	GGCATTTTCTCCTGGCTGTTTTACGC
Mussel_Casp-8_RACE5-1.1	CCACGCAGTTCGGGTTGTATTTTGG

### Nucleotide sequence and structural analysis

ChromasPro (v.1.33) and sCAP3 software packages were used for consensus alignment for the nucleotide sequences. Protein sequences were obtained by using the ExPASy Proteomics Server tools. The consensus sequences were compared with available sequences in the GenBank database using ClustalW2 software. The pTARGET software was used to predict the topology of caspase proteins. The predictions of signal peptides, cleavage sites and the trans-membrane regions were carried out using applicable available software from the ExPASy Proteomics Server (SignalIP 3.0, SOSUI and SMART programs, respectively). The PROSITE database described the protein domains, families and functional sites.

### Phylogenetic analysis

Phylogenetic trees were constructed using the six complete sequences homologous to the caspase proteins previously described and 63 additional caspase sequences from different animals downloaded from Genbank. Protein sequences were aligned with ClustalW2 and two Neighbor-joining (NJ) phylogenetic trees were estimated using MEGA 3 software. Nodal support was estimated using the same program with 10000 bootstrap replicates.

### Tissue expression analysis

The expression of the six caspase genes in different tissues was evaluated using qPCR. Nine naïve mussels were sampled and the foot, gills, gland, female gonad, male gonad, hemocytes, mantle, muscle and palps were removed. Total RNAs were isolated using the Trizol reagent, (Invitrogen) following the manufacturer’s instructions, and mixed (3 individuals per sample). First-strand cDNAs were synthesized with SuperScript III (Invitrogen) using 1 µg of total RNA, treated with Turbo DNA-*free* (Ambion) to remove contaminating DNA. Specific primers were designed with Primer 3 software (v. 0.4.0) and checked to ensure similar efficiencies in the amplification reaction according to the protocol described by Livak and Schmittgen [64] (Table 3). A total of 0.5 µl of each primer (10 µM) was mixed with 10.5 µl of SYBR green PCR master mix (Applied Biosystems) in a final volume of 21 µl. The standard cycling conditions were 95°C for 10 min, followed by 40 cycles of 95°C 15 s and 60°C for 1 min. The comparative Ct method (2^-ΔΔCt^ method) was used to determine the expression level of analyzed genes [64]. The expression of caspase genes was normalized using the β-actin gene as a control housekeeping gene. Fold units were calculated by dividing the normalized expression values in the different tissues by the normalized expression values obtained in the muscle. Data were analyzed using the Student’s t-test. Results were expressed as the mean ± standard deviation of the three different samples and differences were considered statistically significant at p<0.05.

**Table 3:** Sequences of specific primers used for qPCR assays.

Name	Sequence
Mussel_Casp-2_qPCR-4F	GATATATGACAAGGGTGGCAATG
Mussel_Casp-2_qPCR-4R	GACTTTACAGCATCCAGGACATC
Mussel_Casp-3/7-1_qPCR-F	GATCTTGGAAGTGGTGTAGAACG
Mussel_Casp-3/7-1_qPCR-R	CACTGCTAGGAAATCTGCTTCAT
Mussel_Casp-3/7-2_qPCR-3F	CCTGTAGAGGAGGAGGATTAGGAC
Mussel_Casp-3/7-2_qPCR-3R	GGAAGGATCCATAACCAGCAG
Mussel_Casp-3/7-3_qPCR-F	CAATGTGTAAAAACGAGAGACATTG
Mussel_Casp-3/7-3_qPCR-R	GTTAGTATATGCCCACTGTCCATTC
Mussel_Casp-3/7-4_qPCR-2F	GATTTCCTTGAAGTCTTTTCTACGTC
Mussel_Casp-3/7-4_qPCR-2R	AATATCATCGTCTTCCTGGATGTTAT
Mussel_Casp-8_qPCR-F	CCCAACCAGTAGTAACACCAGAC
Mussel_Casp-8_qPCR-R	GTATGAACCATGCCCCTATATCA
Mussel_Actin_qPCR-F	AACCGCCGCTTCTTCATCTTC
Mussel_Actin_qPCR-R	TACCACCAGACAAGACGG

### Apoptosis induction by UV treatment

Hemolymph was collected from the adductor muscle of 9 adult mussels, adjusted to 2×10^6^ cells/ml and placed into 24-well tissue culture plates (Falcon). After 15 min of incubation at 15°C, the volume was reduced to 500 µl and the cells were exposed to UV light (Sylvana G8T5 light bulb) for 45 min. Subsequently, the volume in each well was restored to 1 ml with filtered sterile sea water (FSW) and plates were incubated at 15°C. In all experiments, a control group, not exposed to UV light, was included. After 0, 1, 3, 6 and 24 h post treatment (pt) hemocytes were collected and processed to analyze different aspects of the apoptotic process.

#### Evaluation of the apoptotic process by optical microscopy

Optical microscopy was used to evaluate the morphological changes induced in UV-treated hemocytes. Briefly, hemocytes exposed to UV were detached from the bottom of the plates with a cell scraper (Falcon) and loaded into cytospin cuvettes (Cytospin 4, Thermo Scientific). Samples were applied to glass slides by centrifugation at 28×g for 5 min with low acceleration. Cytospin preparations were allowed to air-dry for 5 min and then stained with Hemacolor kit (Merck). In all cases, the control group was processed with the same protocol. The morphological changes were evaluated in a Nikon Eclipse 80i microscope.

#### Evaluation of the apoptotic process by flow cytometry

The evolution of the apoptotic levels was analyzed by flow cytometry. UV-treated and non-treated hemocytes were collected and transferred to a 1.5 ml Eppendorf. Samples were rinsed twice with PBS and resuspended in 1X binding buffer (10 mM Hepes/NaOH, pH 7.4, 140 mM NaCl, 2.5 mM CaCl_2_) containing 5 µl of Annexin V-FITC (BD Biosciences) and 10 µl of 7-amino-actinomycin D (7-AAD, BD Biosciences). Cells were incubated for 15 min at room temperature in the dark and analyzed in a FACSCalibur flow cytometer using Cell Quest software (BD Biosciences). The percentage of apoptotic and necrotic cells were quantified by using the FITC and 7-AAD content detected in the FL-1 (530 nm) and FL-3 (>650 nm) channels, respectively. Fold units were calculated by dividing the percentage of FL-1 positive hemocytes obtained after UV treatment by the values recorded in the control group. Data were analyzed using the Student’s t-test. Results were expressed as the mean ± standard deviation of the 9 hemocyte samples and differences were considered statistically significant at p<0.05.

#### Modulation of caspase gene expression by UV treatment

In order to determine and quantify the caspase gene expression patterns, real time PCR was performed using RNAs from hemocytes exposed to UV light as previously described. Amplification was carried out using specific primers and the qPCR conditions as described above. The expression levels of the caspase genes were normalized using the β-actin gene as a housekeeping gene. Fold units were calculated by dividing the normalized expression values in the UV-treated hemocytes by the normalized expression values obtained in the control non-treated group. Data were analyzed using the Student’s t-test. Results were expressed as the mean ± standard deviation of 3 pools (3 individual RNAs per pool) and differences were considered statistically significant at p<0.05.

### Modulation of the caspase genes expression under different stimuli

Several PAMPs were added to hemocyte primary cultures. Solutions of 1 mg/ml of poly I:C, zymosan, lipopolysaccharide (LPS) or lipoteichoic acid (LTA) were prepared from a commercial stock (Sigma). To prepare CpG, bacteria (*V. anguillarum*) were grown in TSA supplemented with 1% NaCl at room temperature. Bacterial DNA was then isolated using a phenol-chloroform protocol and CpG concentration was adjusted to 1 mg/ml. Hemolymph was collected from the adductor muscle of 5 naïve individuals and pooled into 4 pools (2.5 ml of hemolymph per pool). Hemocytes (20 primary cultures) were then incubated with the PAMP solutions at a final concentration of 50 µg/ml. Samplings were performed after 1, 3 and 6 h pt. All experiments were performed at 15°C and replicated at least twice.

In order to analyze the effect of environmental pollutants on the mussel immune system, primary cultures of hemocytes were exposed to different PAHs and PCBs (Sigma, Aldrich). Briefly, hemolymph from 12 mussels was collected from the adductor muscle and pooled into 4 samples (3 individuals per sample). Cells were adjusted to a final concentration of 2.5×10^4^ cells/ml with FSW and placed into 24-well tissue culture plates (Falcon). Hemocytes were incubated with PHAs (phenanthrene and benzopyrene) at a final concentration of 100 ppb and with PCBs at 3.5 µg/ml. Control groups were treated with the same volume of FSW. Samplings were performed after 1, 3 and 6 h pt.

Both hemocytes treated with PAMPs and pollutants were processed and analyzed by real time PCR as described above.
